# Sorafenib Promotes Treg Cell Differentiation To Compromise Its Efficacy via VEGFR/AKT/Foxo1 Signaling in Hepatocellular Carcinoma

**DOI:** 10.1016/j.jcmgh.2024.101454

**Published:** 2024-12-30

**Authors:** Yingying Shen, Hanliang Wang, Zeyu Ma, Minyan Hao, Shuowang Wang, Junwei Li, Yue Fang, Lei Yu, Yingying Huang, Changrong Wang, Jingjing Xiang, Zhijian Cai, Jianli Wang, Hongchuan Jin, Jia Zhou, Jufeng Guo, Pingting Ying, Xian Wang

**Affiliations:** 1 Department of Medical Oncology, Zhejiang Key Laboratory of Multi-omics Precision Diagnosis and Treatment of Liver Diseases, Cancer Center of Zhejiang University, Sir Run Run Shaw Hospital, Medical School of Zhejiang University, Hangzhou, Zhejiang, China; 2 Department of Dermatology and Venerology, Sir Run Run Shaw Hospital, Medical School of Zhejiang University, Hangzhou, Zhejiang, China; 3 Institute of Immunology and Department of Orthopaedics of the Second Affiliated Hospital, Zhejiang University School of Medicine, Hangzhou, Zhejiang, China; 4 Core Facilities, School of Medicine Zhejiang University, Hangzhou, China; 5 Department of Pathology, Affiliated Hangzhou First People’s Hospital, School of Medicine, Westlake University, Hangzhou, Zhejiang, China; 6 Institute of Immunology and Bone Marrow Transplantation Center of the First Affiliated Hospital, Zhejiang University School of Medicine, Hangzhou, Zhejiang, China; 7 Institute of Hematology, Zhejiang University & Zhejiang Engineering Laboratory for Stem Cell and Immunotherapy, Hangzhou, Zhejiang, China; 8 Department of Breast Surgery, Affiliated Hangzhou First People’s Hospital, School of Medicine, Westlake University, Hangzhou, Zhejiang, China

**Keywords:** Hepatocellular Carcinoma, Sora Resistance, Treg Cells, Foxo1, Immunosuppression

## Abstract

**Background & Aims:**

Sora is the first-line drug for advanced hepatocellular carcinoma (HCC). However, acquired resistance to Sora treatment largely hinders its therapeutic efficacy, and the mechanisms underlying Sora resistance remain poorly understood. Here, we revealed a new mechanism by which Sora promotes the differentiation of regulatory T (Treg) cells to suppress the immune response in the HCC tumor microenvironment (TME) and induce Sora resistance.

**Methods:**

Human liver tissues were obtained from HCC patients. Female C57BL/6J, OT-II, and *Foxp3*^*GFP*^ mice were also used. Flow cytometry was used to analyze immune cells in TME. Flow cytometry, real-time polymerase chain reaction, and enzyme-linked immunosorbent assay were performed to evaluate Treg cell differentiation. Immunoblotting was conducted to identify relevant proteins. Mouse and human tumor tissues were evaluated via multiplex immunofluorescence staining. Sora-treated HCC tissues and Sora-treated Treg cells were subjected to RNA sequencing analysis. Tumor models were generated and treated with Sora, Sora combined with an anti-CD25 antibody, or Sora combined with the Foxo1 inhibitor AS1842856.

**Results:**

First, we found through bioinformatic analysis that Sora suppresses the immune response in HCC. Furthermore, Sora increased the Treg cell population to promote the formation of an immunosuppressive TME in HCC. In vitro, Sora promoted Treg cell differentiation and increased the immunosuppressive activity of Treg cells. Activating VEGF and AKT abolished the effect of Sora on Treg cell differentiation, whereas inhibiting Foxo1 compromised Sora-induced Treg cell differentiation, indicating that the induction of Treg cells by Sora is dependent on the VEGFR/AKT/Foxo1 pathway. Finally, Treg inactivation by an anti-CD25 antibody or the Foxo1 inhibitor AS1842856 in combination with Sora showed greater efficacy in the treatment of HCC.

**Conclusions:**

Sora induced Treg cell differentiation by inhibiting VEGFR/AKT signaling and activating Foxo1, thus suppressing the immune response and reducing Sora efficacy. Treg inactivation might be a promising strategy to alleviate the immunosuppressive TME and overcome Sora resistance.

## Graphical abstract

**Figure:**
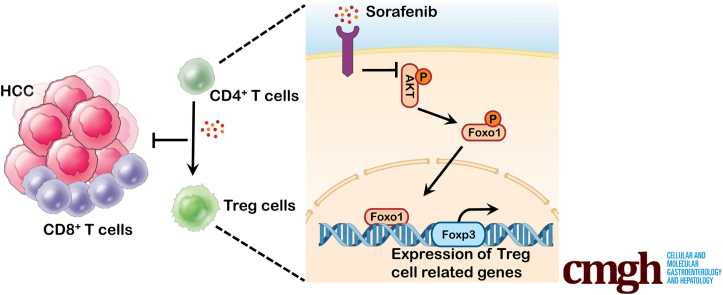

**Summary**

Our study revealed that sorafenib (Sora) induced the formation of an immunosuppressive tumor microenvironment in hepatocellular carcinoma (HCC) by promoting the differentiation of regulatory T (Treg) cells through VEGFR/AKT/Foxo1 signaling, leading to compromised Sora efficacy. Importantly, combination treatment with an anti-CD25 antibody or the Foxo1 inhibitor AS1842856 inhibited Treg cell differentiation and increased the therapeutic efficacy of Sora in HCC.

This article has an accompanying editorial.

Hepatocellular carcinoma (HCC) is the sixth most common carcinoma and the third leading cause of cancer-related deaths globally, despite significant progress in HCC treatment in recent years.^1^ Approximately 60% of HCC patients are diagnosed at an advanced stage (Barcelona Clinic Liver Cancer stage B or higher).^2^ Sorafenib (Sora) is the first Food and Drug Administration–approved targeted drug for advanced HCC.^3^, ^4^, ^5^ As a multiple-target kinase inhibitor, Sora suppresses tumor cell proliferation by inhibiting the activity of the intracellular serine/threonine kinases Ras/Raf/MEK/ERK and blocks tumor angiogenesis by targeting the cell surface tyrosine kinase receptors VEGFR, PDGFR, c-KIT, and FLT-3.^6^^,^^7^ Although Sora can increase the survival of HCC patients, its efficacy is limited by the rapid development of Sora resistance. Several studies have suggested that the genetic and epigenetics changes in HCC cells play an important role in the initiation and development of Sora resistance in HCC patients.^8^^,^^9^

Recently, tumor microenvironment (TME) remodeling was found to play an important role in the acquirement of Sora resistance in HCC cells. For example, tumor-associated M2 macrophages mediate the resistance of HCC cells to Sora by activating the c-Met/MAPK and PI3K/AKT signaling pathways.^10^ In addition, tumor-associated N2 neutrophils can recruit the FoxP3^+^CD25^+^CD4^+^ T lymphocyte subset to increase Sora resistance in HCC cells.^11^, ^12^, ^13^ Moreover, myeloid-derived suppressor cells (MDSCs) facilitate CAF activation and subsequent Sora resistance by inducing FGF1 expression.^14^

In this study, we showed that Sora promotes the differentiation but not the proliferation of immunosuppressive regulator T (Treg) cells both in vitro and in vivo by modulating the VEGFR/Akt/Foxo1 pathway in CD4^+^ T cells. Inhibiting Treg cell differentiation with an anti-CD25 antibody or the Foxo1 inhibitor AS1842856 increased the efficacy of Sora in the treatment of HCC in vivo.

## Results

### Sora Tends to Induce Immune Suppression in HCC

**Figure 1:**
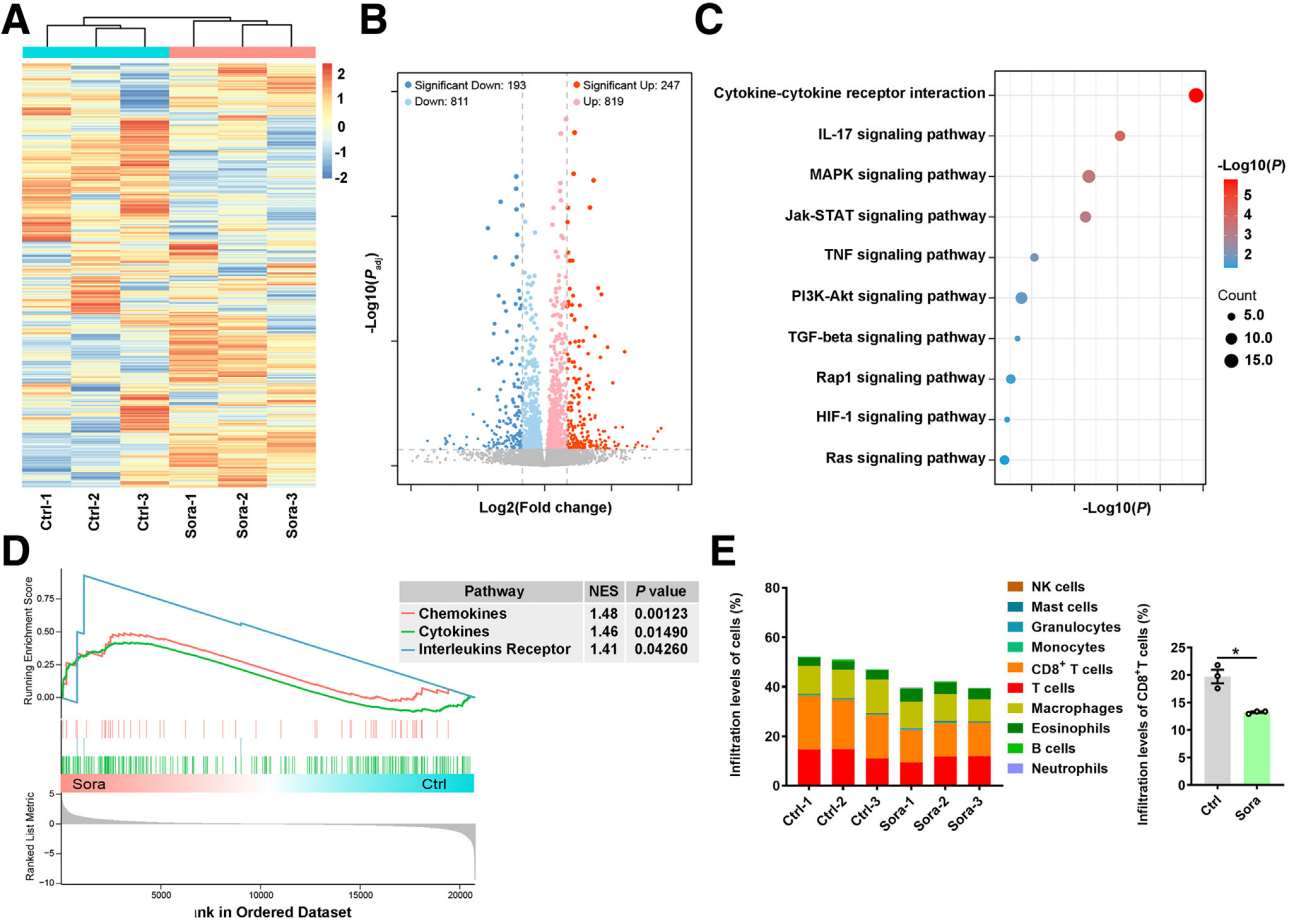
**Sora suppresses the immune response in HCC.** (*A*) HCC tumors were collected from Hepa1-6 tumor-bearing mice that received ip injection of 10 mg kg^−1^ Sora on days 9, 11, 13, and 15. Heatmap of RNA-seq data from control HCC tumors and Sora-treated HCC tumors. (*B*) Scatterplot of RNA-seq data showing genes whose expression was up-regulated (*red dots*) or down-regulated (*blue dots*) by at least 2-fold in Sora-treated HCC tumors relative to control HCC tumors and genes with similar expression in HCC tumors treated with or without Sora (*gray dots*). Differential expression analysis was performed using the DESeq2 package in R with default parameters. |log_2_ FoldChange|>1 and *P*_adj_ <.05 were used to obtain a final list of DEGs. (*C*) Gene ontology molecular function analysis is shown, with KEGG pathways enriched in control HCC tumors and Sora-treated HCC tumors. (*D*) Enrichment of the chemokine, cytokine, and interleukin receptor pathways according to GSEA in control HCC tumors and Sora-treated HCC tumors. (*E*) The number of DEGs associated with immune cell infiltration in control and Sora-treated HCC tumors was estimated via mMCPcounter. ∗*P* < .05.

To explore the gene expression changes in HCC tumor tissues after Sora treatment, we analyzed the expression profiles of mouse HCC tumor tissues with or without Sora treatment through whole-transcriptome RNA-sequencing (RNA-seq). Compared with control mouse HCC tumors, Sora-treated mouse HCC tumors presented 819 up-regulated genes and 811 down-regulated genes (Figure 1*A* and *B*). Through pathway enrichment analysis, we found that these differentially expressed genes (DEGs) were associated with multiple signaling pathways (Figure 1*C*). Gene-set enrichment analysis (GSEA) of the RNA-seq data revealed that chemokine and cytokine signaling and interleukin receptor signaling were increased in Sora-treated HCC tumors (Figure 1*D*). We further analyzed the associations between the expression levels of DEGs and immune cell infiltration and found that these genes were closely related to immune cell infiltration. Among the DEGs in the Sora-treated HCC tumor, those associated with CD8^+^ T cells were down-regulated (Figure 1*E*).^15^ Together, these results indicate that Sora may suppress the immune response in the HCC TME.

### Sora Promotes Treg Cell Differentiation in HCC

**Figure 2:**
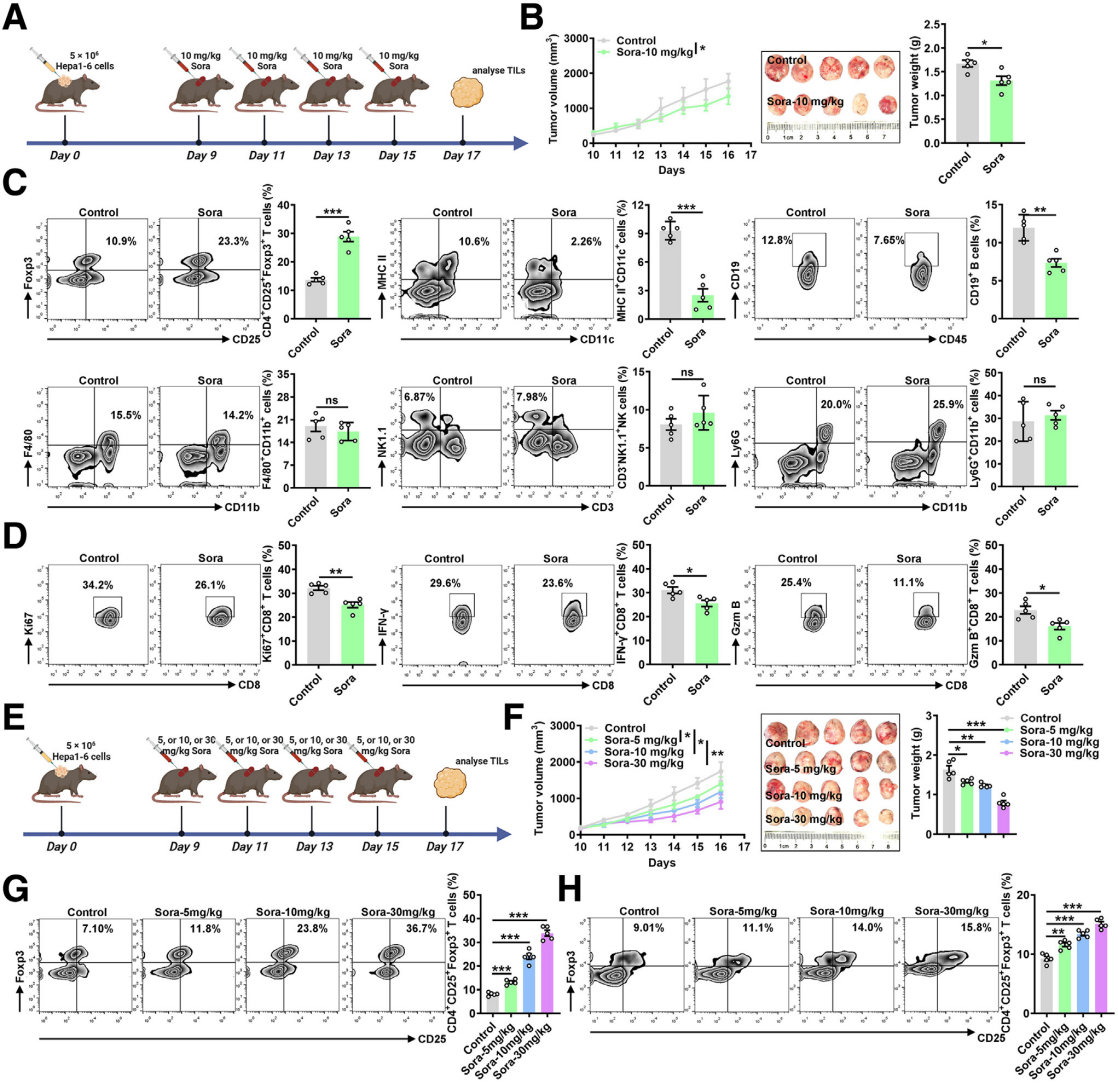
**Sora increases Treg cell activity in HCC.** (*A*) Schematic of murine model established with Hepa1-6 liver cancer cells and time points at which Sora was administered via ip injection. (*B*) Tumor sizes (*left*) in Hepa1-6 tumor-bearing mice that received ip injection of 10 mg kg^−1^ Sora was recorded every other day, and the tumor weights (*right*) were measured immediately after they were excised and plotted. (*C* and *D*) FC analysis of CD4^+^CD25^+^Foxp3^+^ Treg cells, MHC-II^+^CD11c^+^ DCs, CD19^+^ B cells, F4/80^+^CD11b^+^ macrophages, CD3^-^NK1.1^+^ NK cells, Ly6G^+^CD11b^+^ neutrophils (*C*), and Ki-67^+^CD8^+^ T, IFN-γ^+^CD8^+^ T, and granzyme B^+^CD8^+^ T cells (*D*) among TILs on day 17 in Hepa1-6 tumor-bearing mice that received ip injection of 10 mg kg^−1^ Sora every 2 days (*left*) and the corresponding statistical analysis (*right*). (*E*) Schematic of murine model established with Hepa1-6 liver cancer cells and time points at which Sora was administered via ip injection. (*F*) Tumor sizes (*left*) in Hepa1-6 tumor-bearing mice that received ip injections of 5, 10, or 30 mg kg^−1^ Sora were recorded every other day, and the tumor weights (*right*) were measured immediately after they were excised and plotted. (*G* and *H*) FC analysis of CD4^+^CD25^+^Foxp3^+^ Treg cells among TILs (*G*) or in the spleen (*H*) on day 17 in Hepa1-6 tumor-bearing mice that received ip injections of 5, 10, or 30 mg kg^−1^ Sora every 2 days (*left*) and the corresponding statistical analysis (*right*). Representative results from 3 independent experiments are shown (mean ± SD) (n = 3). ∗*P* < .05; ∗∗*P* < .01; ∗∗∗*P* < .001; ns, not significant (unpaired Student *t* test).

Next, we established mouse HCC tumor model to validate the effect of Sora on the tumor immune microenvironment comprehensively. Sora inhibited tumor progression in a murine model established with Hepa1-6 liver cancer cells (Figure 2*A*) and was cytotoxic to Hepa1-6 cells (Figure 2*B*). Interestingly, Sora significantly increased the proportions of CD4^+^CD25^+^Foxp3^+^ Treg cells among tumor-infiltrating leukocytes (TILs) but decreased the proportions of MHC-II^+^CD11c^+^ dendritic cells (DCs) and CD19^+^ B cells among TILs (Figure 2*C*), suggesting that Sora treatment led to the formation of an immunosuppressive TME. However, Sora had no effect on F4/80^+^CD11b^+^ macrophages, CD3^-^NK1.1^+^ natural killer (NK) cells, or Ly6G^+^CD11b^+^ neutrophils among TILs (Figure 2*C*). In addition, consistent with the increase in the proportion of Treg cells, Sora also decreased the levels of Ki-67, IFN-γ, and granzyme B in CD8^+^ T cells (Figure 2*D*), suggesting the inhibition of T-cell activation.

To further confirm the effect of Sora in Treg cells in HCC, we treated tumor-bearing mice with Sora at doses of 5, 10, or 30 mg kg^-1^ (Figure 2*E*), and Sora inhibited the growth of liver cancer in a dose-dependent manner (Figure 2*F*). We detected a concentration-dependent increase in the proportion of Treg cells among TILs in Sora-treated tumor-bearing mice (Figure 2*G*). In addition, an increase in the number of Treg cells in the spleen was also observed (Figure 2*H*). Thus, Sora suppresses the immune response in HCC by increasing the Treg cell population in the TME.

### Sora Promotes Treg Cell Differentiation in Vitro

**Figure 3:**
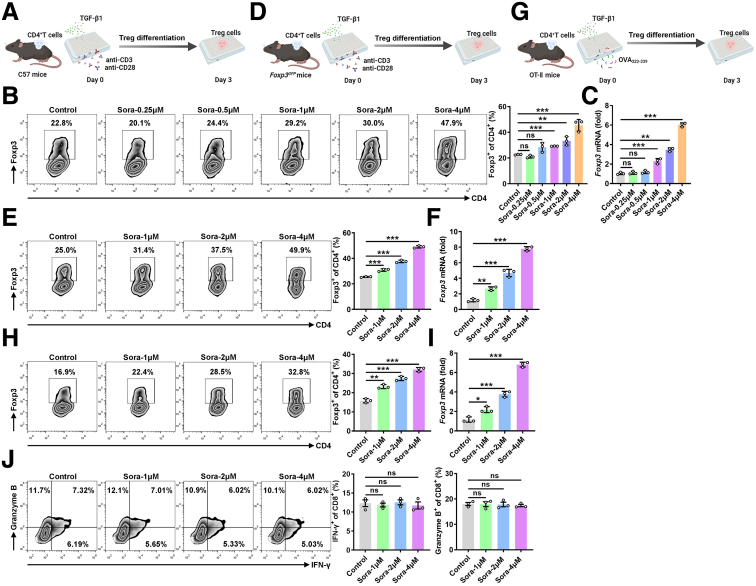
**Sora promotes Treg cell induction in vitro.** (*A*) Schematic of in vitro induction of Tregs isolated from C57BL/6J mice. (*B* and *C*) FC analysis of Foxp3^+^CD4^+^ T cells (*left*) and the corresponding statistical analysis (*right*) (*B*), real-time PCR analysis of *Foxp3* mRNA expression (*C*) in naive CD4^+^ T cells stimulated with anti-CD3 antibodies, anti-CD28 antibodies, and the indicated concentrations of Sora under Treg-skewing conditions for 4 days. (*D*) Schematic of the in vitro induction of Tregs isolated from *Foxp3*^*GFP*^ mice. (*E* and *F*) FC analysis of GFP^+^CD4^+^ T cells (*left*) and the corresponding statistical analysis (*right*) (*E*), real-time PCR analysis of *Foxp3* mRNA expression (*F*) in naive CD4^+^ T cells from *Foxp3*^*GFP*^ mice stimulated with anti-CD3 or anti-CD28 antibodies in the presence of 1, 2, or 4 μmol/L Sora under Treg-skewing conditions for 4 days. (*G*) Schematic of the in vitro induction of Tregs isolated from OT II mice. (*H* and *I*) FC analysis of Foxp3^+^CD4^+^ T cells (*left*) and the corresponding statistical analysis (*right*) (*H*), real-time PCR analysis of *Foxp3* mRNA expression (*I*) in naive CD4^+^ T cells from OT-II mice stimulated with 2 μg mL^−1^ OVA_323–339_ peptide plus T-cell-depleted γ-irradiated splenic cells in the presence of 1, 2, or 4 μmol/L Sora and 10 ng mL^-1^ TGF-β1 for 4 days. (*J*) CD8^+^ T cells were sorted from C57BL/6J mice and stimulated with anti-CD3 or anti-CD28 antibodies in the presence of Sora for 24 hours. FC analysis of IFN-γ and granzyme B levels in CD8^+^ T cells (*left*) and the corresponding statistical analysis (*right*). Representative results from 3 independent experiments are shown (mean ± SD) (n = 3). ∗*P* < .05; ∗∗*P* < .01; ∗∗∗*P* < .001; ns, not significant (unpaired Student *t* test).

To further confirm the function of Sora in Treg cell differentiation, we differentiated naive CD4^+^ T cells from C57BL/6 mice into Treg cells via T-cell receptor (TCR) stimulation combined with TGF-β1 cytokine treatment (Figure 3*A*). We found that low concentrations of Sora (0.25 and 0.5 μmol/L) did not affect Treg cell differentiation (Figure 3*B*). However, high concentrations of Sora (1, 2, and 4 μmol/L) strongly promoted Treg cell differentiation (Figure 3*B*). The results of real-time polymerase chain reaction (PCR) also confirmed that Sora promoted Treg cell differentiation (Figure 3*C*). Moreover, *Foxp3*^*GFP*^ knock-in C57BL/6 mice were generated by inserting the *GFP* gene into the endogenous *Foxp3* locus.^16^ We subsequently differentiated naive CD4^+^ T cells isolated from *Foxp3*^*GFP*^ transgenic mice into Treg cells and treated with the Sora (Figure 3*D*). Similarly, Sora (1, 2, and 4 μmol/L) promoted Treg cell differentiation in TCR-stimulated CD4^+^ T cells from *Foxp3*^*GFP*^ transgenic mice (Figure 3*E* and *F*). Next, we established an antigen-stimulation differentiation model using naive CD4^+^ T cells from OT II transgenic mice in which OT II CD4^+^ T cells transgenically express a TCR that recognizes an epitope of OVA_323–339_ in the context of I-Ab^17^ (Figure 3*G*). Sora facilitated the differentiation of CD4^+^ T cells from OT-II transgenic mice into Treg cells in response to their cognate antigen OVA_323–339_ (Figure 3*H* and *I*). In addition, Sora did not directly influence the levels of IFN-γ and granzyme B in CD8^+^ T cells directly (Figure 3*J*). Taken together, these results indicate that Sora promotes the induction of Treg cells in vitro.

### Sora Induces a Suppressive and Activated Phenotype in Treg Cells in Vitro

**Figure 4:**
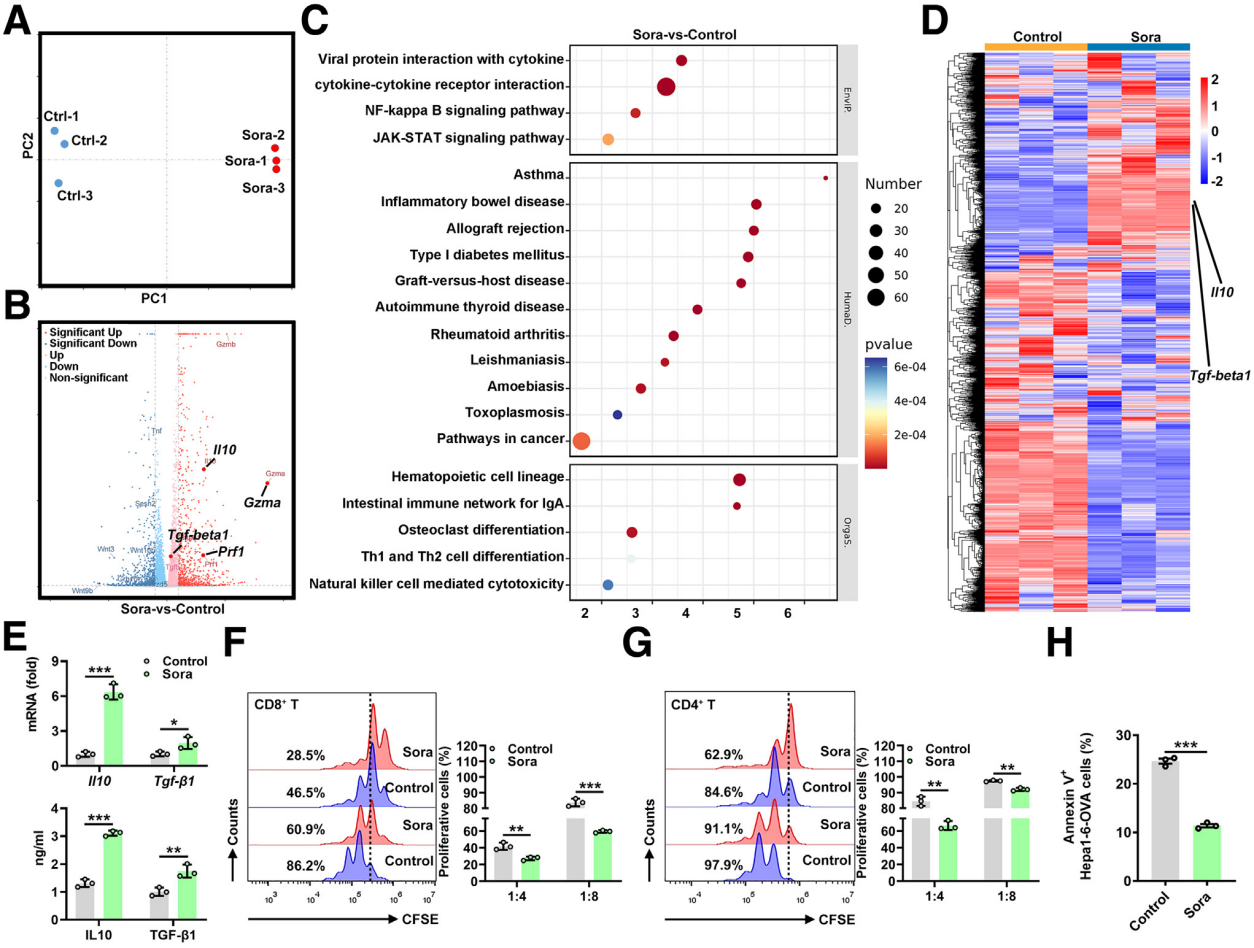
**Sora promotes an immunosuppressive and activated Treg cell phenotype.** (*A*) Principal component analysis (PCA) plots based on all expressed genes in control-Treg cells and Sora-Treg cells. (*B*) Scatterplot of RNA-seq data showing genes whose expression was up-regulated (*red dots*) or down-regulated (*blue dots*) by at least 2-fold in Sora-Treg cells relative to that in control-Treg cells and genes with similar expression in Sora-Treg cells and control-Treg cells (*gray dots*). (*C*) Gene ontology molecular function analysis is shown, with enriched KEGG pathways in Sora-Treg cells and control-Treg cells. (*D*) Heatmap of RNA-seq data from Treg cells for each sample in the control and Sora-Treg groups. (*E*) Real-time PCR (*top*) and ELISA (*bottom*) analysis of *Il10* and *Tgf-beta1* mRNA and protein expression in control-Treg cells or Sora-Treg cells differentiated from naive CD4^+^ T cells from *Foxp3*^*GFP*^ transgenic mice stimulated with anti-CD3 or anti-CD28 antibodies in the presence of 4 μmol/L Sora under Treg-skewing conditions for 4 days. (*F* and *G*) FC analysis of the proliferation of CFSE-labeled CD8^+^ (*F*) or CD4^+^ T cells (*G*) cocultured with control-Treg cells or Sora-Treg cells differentiated from naive CD4^+^ T cells from C57BL/6J mice stimulated with anti-CD3 or anti-CD28 antibodies in the presence of 4 μmol/L Sora under Treg-skewing conditions for 4 days and the corresponding statistical analysis. Cells were cultured at T cell: Treg cell ratio of 4:1 or 8:1 in anti-CD3- or anti-CD28-coated plates for 5 days. (*H*) CD8^+^ T cells in (*F*) were sorted and cocultured with Hepa1-6-OVA cells at a CD8^+^ T cell: Hepa1-6-OVA cell ratio of 10:1. FC analysis of Annexin V^+^ Hepa1-6-OVA cells. Representative results from 3 independent experiments are shown (mean ± SD) (n = 3). ∗*P* < .05; ∗∗*P* < .01; ∗∗∗*P* < .001 (unpaired Student *t* test).

To obtain a comprehensive view of how Sora regulates the Treg cell phenotype, we performed RNA-seq. We differentiated naive CD4^+^ T cells from *Foxp3*^*GFP*^ transgenic mice into Treg cells with or without Sora in vitro and subsequently sorted GFP^+^ Treg cells for RNA-seq. There were many DEGs between the ctrl-Treg cells and Sora-Treg cells (Figure 4*A* and *B*). Focusing on genes associated with immunosuppressive function, we noted that the expression of Treg effector genes such as *Il10*, *Prf1,* and *Gzma* was increased in Sora-Treg cells (Figure 4*B*). In addition, Kyoto Encyclopedia of Genes and Genomes (KEGG) analysis revealed global transcriptomic changes in pathways associated with environmental information processing (cytokine-cytokine receptor interaction), organismal systems (pathways in cancer), and human diseases (hematopoietic cell lineage) (Figure 4*C*). A heatmap obtained via unsupervised hierarchical clustering revealed that *Il10* and *Tgf-beta1* were up-regulated in Sora-Treg cells compared with ctrl-Treg cells (Figure 4*D*), which was confirmed by real-time PCR and enzyme-linked immunosorbent assay (ELISA) (Figure 4*E*), suggesting that Sora-Treg cells presented an immunosuppressive and activated phenotype.

To further confirm that Sora increased the suppressive capacity of Treg cells, we used a proliferation suppression assay with T cells. CD4^+^ T or CD8^+^ T cells were cocultured with ctrl-Treg cells or Sora-Treg cells. We found that Sora-Treg cells significantly decreased the proliferation of CD8^+^ T cells (Figure 4*F*), which was also observed in CD4^+^ T cells (Figure 4*G*). Furthermore, compared with CD8^+^ T cells treated with ctrl-Treg cells, CD8^+^ T cells treated with Sora-Treg cells were less cytotoxic to Hepa1-6-OVA cells (Figure 4*H*). Together, these findings indicate that Sora induces Treg cells to possess an immunosuppressive and activated phenotype.

### Sora Induces Treg Cell Differentiation via VEGFR/AKT/Foxo1 Signaling

**Figure 5:**
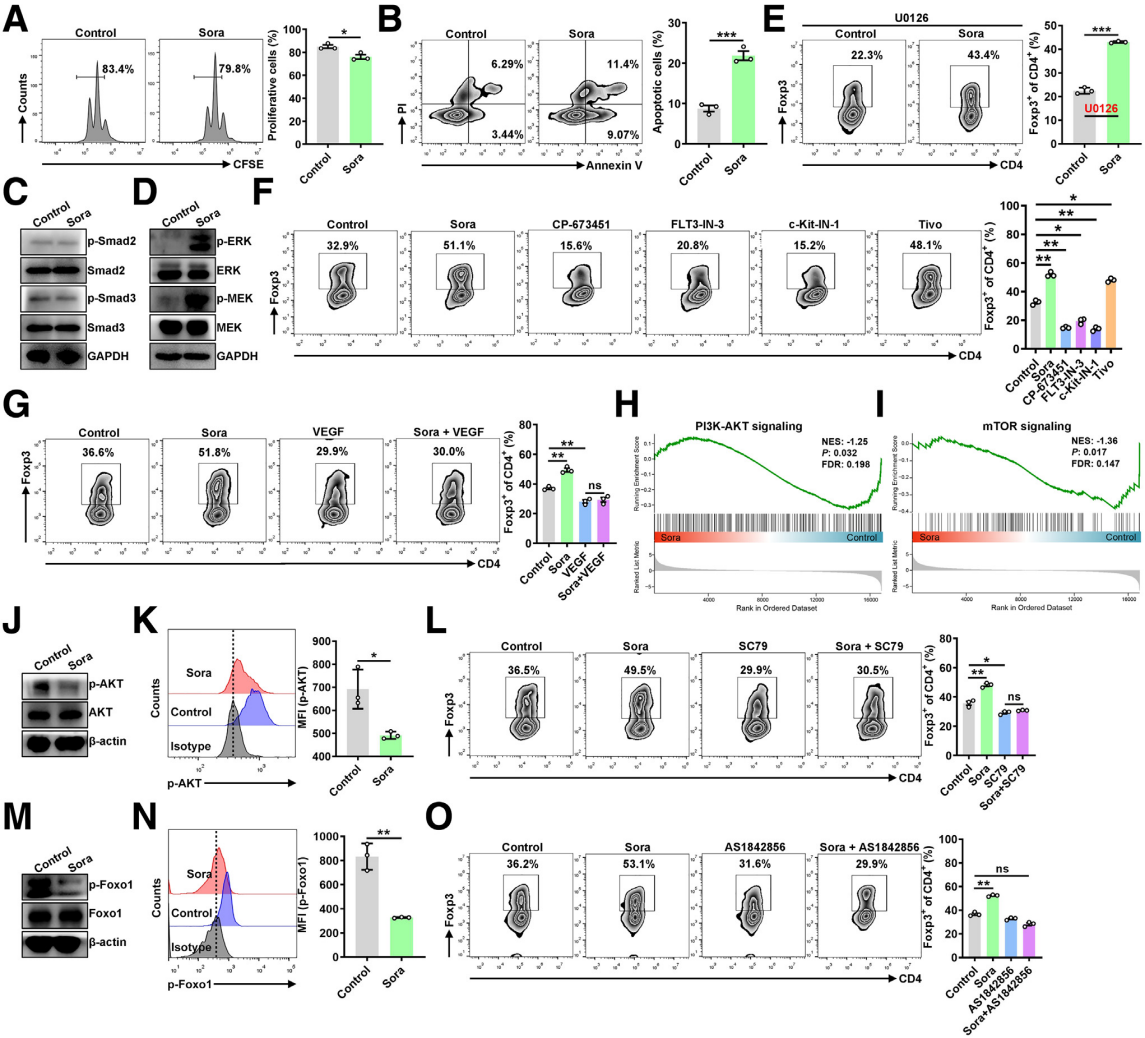
**Sora induces Treg cell differentiation via VEGFR/AKT/Foxo1 signaling.** (*A* and *B*) Naive CD4^+^ T cells from C57BL/6J mice with (*A*) or without (*B*) CFSE labeling were differentiated into Treg cells under Treg-skewing conditions with or without 4 μmol/L Sora for 3 days. FC analysis of Treg proliferation, as measured by CFSE dilution and the corresponding statistical analysis (*A*), and FC analysis of apoptotic cells and the corresponding statistical analysis (*B*). (*C* and *D*) Immunoblot analysis of the indicated proteins in naive CD4^+^ T cells in the presence of 4 μmol/L Sora under Treg-skewing conditions for 24 hours. (*E*) FC analysis of Foxp3^+^CD4^+^ T cells in naive CD4^+^ T cells treated with 0.5 μmol/L U0126 or 4 μmol/L Sora under Treg-skewing conditions for 4 days. (*F* and *G*) FC analysis of Foxp3^+^CD4^+^ T cells in naive CD4^+^ T cells treated with 0.5 μmol/L CP-673451, 0.5 μmol/L FLT3-IN-3, 0.5 μmol/L c-Kit-IN-1, 1 μmol/L Tivo (*F*), 10 ng/mL VEGF (*G*), or 4 μmol/L Sora (*F* and *G*) under Treg-skewing conditions for 4 days (*left*) and the corresponding statistical analysis (*right*). (*H* and *I*) Enrichment of the PI3K-AKT signaling pathway (*H*) and the mTOR signaling pathway (*I*) according to GSEA in control-Treg cells and Sora-Treg cells. (*J* and *K*) Immunoblot analysis (*J*) and FC analysis (*K*) of the indicated proteins in naive CD4^+^ T cells in the presence of 4 μmol/L Sora under Treg-skewing conditions for 24 hours. (*L*) FC analysis of Foxp3^+^CD4^+^ T cells in naive CD4^+^ T cells treated with 0.1 μmol/L SC79 or 4 μmol/L Sora under Treg-skewing conditions for 4 days (*left*) and the corresponding statistical analysis (*right*). (*M* and *N*) Immunoblot analysis (*M*) and FC analysis (*N*) of p-Foxo1 in naive CD4^+^ T cells in the presence of 4 μmol/L Sora under Treg-skewing conditions for 24 hours. (*O*) FC analysis of Foxp3^+^CD4^+^ T cells in naive CD4^+^ T cells treated with 1 μmol/L AS1842856 or 4 μmol/L Sora under Treg-skewing conditions for 4 days (*left*) and the corresponding statistical analysis (*right*). Representative results from 3 independent experiments are shown (mean ± SD) (n = 3). ∗*P* < .05; ∗∗*P* < .01; ∗∗∗*P* < .001; ns, not significant (unpaired Student *t* test).

Next, we explored how Sora regulates Treg cell differentiation. Sora slightly inhibited the proliferation of Treg cells and promoted their apoptosis (Figure 5*A* and *B*). Because TGF-β1 signaling-activated Smad2 and Smad3 are key transcripts for Treg cell differentiation,^18^^,^^19^ we detected the activation of Smad2 and Smad3 in control and Sora-treated CD4^+^ T cells under Treg-skewing conditions. We found that phosphorylated Smad2 and Smad3 protein levels did not differ between control and Sora-treated Treg cells (Figure 5*C*). In addition, ERK signaling is important for TCR activation, which maintains Treg cell homeostasis.^20^^,^^21^ Indeed, the levels of phosphorylated ERK and MEK proteins were greater in Sora-treated CD4^+^ T cells than in control CD4^+^ T cells (Figure 5*D*). However, inhibition of ERK signaling by U0126 failed to inhibit the Treg differentiation induced by Sora (Figure 5*E*), indicating that other signaling pathways might be important for Sora-induced Treg differentiation.

As a multikinase inhibitor, Sora can also inhibit the activity of VEGFR, PDGFR, FLT3, and c-Kit. We subsequently explored their relevance to Treg differentiation via the use of their inhibitors. Whereas CP-673451 (PDGFR inhibitor), FLT3-IN-3 (FLT3 inhibitor), and c-Kit-IN-1 (c-Kit inhibitor) did not promote Treg cell differentiation, Tivo (VEGFR inhibitor) did (Figure 5*F*), indicating that Sora might promote Treg cell differentiation via VEGFR. Furthermore, VEGF, a VEGFR activator, abolished Sora-induced Treg cell differentiation (Figure 5*G*). GSEA of the RNA-seq data revealed that Sora decreased PI3K/AKT signaling (Figure 5*H*) and mTOR signaling, which is downstream of the VEGFR/PI3K/AKT pathway^22^ (Figure 5*I*). In addition, Sora treatment also decreased the phosphorylation of AKT (Figure 5*J* and *K*). Moreover, the AKT activator SC79 succeeded in inhibiting Sora-induced Treg cell differentiation (Figure 5*L*). Akt phosphorylates and inhibits Foxo1, which is important for regulating *Foxp3* expression and Treg function.^23^^,^^24^ Indeed, we also observed that Sora inhibited the phosphorylation of the Foxo1 protein (Figure 5*M* and *N*). Furthermore, inhibition of Foxo1 by its inhibitor AS1842856 inhibited Sora-induced Treg cell differentiation (Figure 5*O*).

Collectively, our data demonstrate that Sora inhibits VEGFR, which subsequently suppresses Akt and activates Foxo1 to promote Treg cell differentiation.

### Sora Promoted an Immunosuppressive Treg Cell Phenotype in Vivo

**Figure 6:**
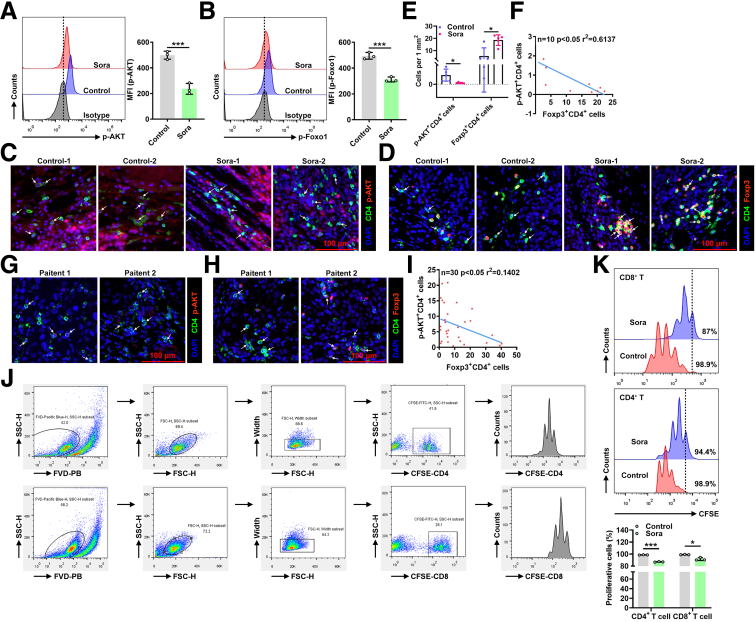
**Sora promotes an immunosuppressive Treg cell phenotype in vivo.** (*A* and *B*) FC analysis of the phosphorylation of the AKT protein (*A*) or the phosphorylation of the Foxo1 protein (*B*) in CD4^+^ T cells among TILs on day 17 in Hepa1-6 tumor-bearing mice that received ip injections of 10 mg kg^-1^ Sora every 2 days (*left*) and the corresponding statistical analysis (*right*). (*C*–*E*) Immunofluorescence analysis of p-AKT (*red*) and CD4^+^ T (*green*) (*C*) or Foxp3 (*red*) and CD4^+^ T (*green*) (*D*) among tumor tissues on day 17 in Hepa1-6 tumor-bearing mice that received ip injections of 10 mg kg^-1^ Sora every 2 days and the corresponding statistical analysis (*E*). (*F*) Pearson correlation between p-AKT^+^CD4^+^ T-cell and Foxp3^+^CD4^+^ T-cell numbers in tumor tissues from Hepa1-6 tumor-bearing mice treated with or without Sora. (*G* and *H*) Immunofluorescence analysis of p-AKT (*red*) and CD4^+^ T (*green*) (*G*) or Foxp3 (*red*) and CD4^+^ T (*green*) (*H*) in human HCC tumor tissues. (*I*) Pearson correlation between p-AKT^+^CD4^+^ T cells and Foxp3^+^CD4^+^ T cells in tumor tissues from human HCC patients. (*J*) The gating strategy for the FC analyses in (*K*). (*K*) FC analysis of the proliferation of CFSE-labeled CD4^+^ or CD8^+^ T cells cocultured with control-Treg cells or Sora-Treg cells from the spleens of Sora-treated Hepa1-6 tumor-bearing mice on day 17 (*left*) and the corresponding statistical analysis (*right*). The cells were cultured at a T-cell: Treg cell ratio of 4:1 in anti-CD3 antibody and anti-CD28 antibody-coated plates for 5 days. Representative results from 3 independent experiments are shown (mean ± SD) (n = 3). ∗*P* < .05; ∗∗*P* < .01; ∗∗∗*P* < .001; ns, not significant (unpaired Student *t* test).

To further explore how Sora regulates Treg cells in TME, we investigated AKT/Foxo1 signaling and confirmed that Sora treatment indeed reduced the phosphorylation of the AKT and Foxo1 proteins via flow cytometric (FC) analysis (Figure 6*A* and *B*). In addition, in situ immunofluorescence staining revealed a decrease in the phosphorylation of the AKT protein in the CD4^+^ T cells isolated from the tumor tissues of Sora-treated mice (Figure 6*C* and *E*). Moreover, immunofluorescence revealed more Treg cells were observed in Sora-treated tumors than in control tumors (Figure 6*D* and *E*). Further analysis confirmed the negative correlation between the numbers of AKT^+^CD4^+^ T cells and Foxp3^+^CD4^+^ T cells in the mouse tumor tissues (Figure 6*F*). Importantly, in human HCC tissues, we also observed the negative correlation between the numbers of AKT^+^CD4^+^ T cells and Foxp3^+^CD4^+^ T cells (Figure 6G–*I*).

Because Sora-induced Treg cells present a more immunosuppressive phenotype in vitro, we examined the function of Treg cells isolated from Sora-treated mice. Compared with Treg cells sorted from control mice, Treg cells isolated from Sora-treated mice had greater immunosuppressive effects (Figure 6*J* and *K*). Taken together, these results indicate that Sora promoted a suppressive and activated Treg cell phenotype in vivo.

### Agents That Inactivate Treg Cells Increase the Therapeutic Efficacy of Sora in HCC

Whereas Sora treatment can inhibit tumor growth, it also promotes Treg cell differentiation, thus suppressing the immune response in the TME and reducing its antitumor efficiency. Therefore, Treg inhibition may be an effective strategy for reactivating the immune system and increasing the therapeutic efficacy of Sora.

**Figure 7:**
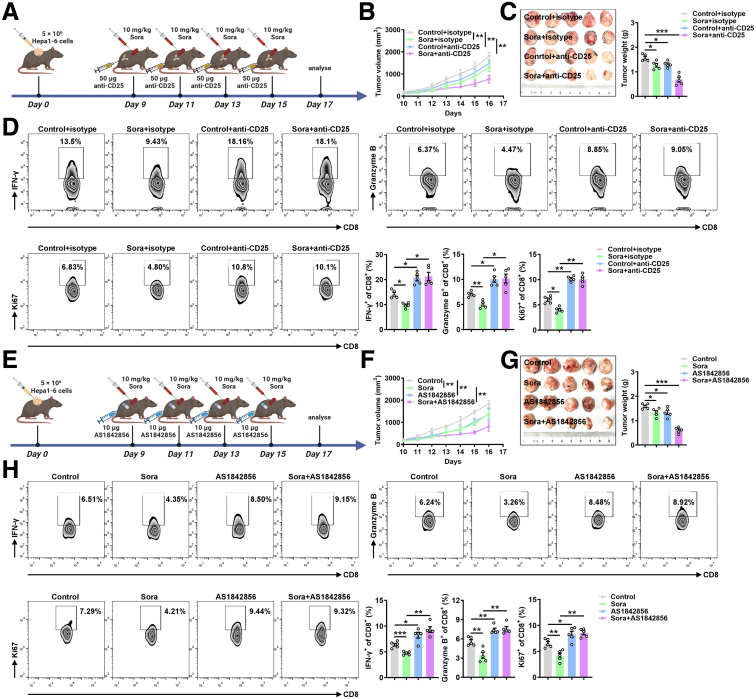
**Agents that deplete Treg cells increase the therapeutic efficacy of Sora.** (*A*) Schematic of the murine model established with Hepa1-6 liver cancer cells with the time points and doses of Sora and the anti-CD25 antibody. (*B* and *C*) Tumor sizes (*B*) in Hepa1-6 tumor-bearing mice that received ip injections of 10 mg kg^−1^ Sora or 50 μg anti-CD25 antibody were recorded every other day, and the tumor weights (*C*) were measured immediately after they were excised and plotted. (*D*) FC analysis of IFN-γ^+^CD8^+^ T cells, granzyme B^+^CD8^+^ T cells, and Ki67^+^CD8^+^ T cells among the spleens of Hepa1-6 tumor-bearing mice that received ip injections of 10 mg kg^−1^ Sora and 50 μg anti-CD25 antibody every 2 days on day 17; the corresponding statistical analysis is shown. (*E*) Schematic of the murine model established with Hepa1-6 liver cancer cells and the time points at which Sora and AS1842856 were injected intraperitoneally. (*F* and *G*) Tumor sizes (*F*) in Hepa1-6 tumor-bearing mice that received ip injections of 10 mg kg^−1^ Sora and 10 mg kg^−1^ AS1842856 were recorded every other day, and the tumor weights (*G*) were measured immediately after they were excised and plotted. (*H*) FC analysis of IFN-γ^+^CD8^+^ T cells, granzyme B^+^CD8^+^ T cells, and Ki67^+^CD8^+^ T cells among the spleens of Hepa1-6 tumor-bearing mice that received ip injections of 10 mg kg^−1^ Sora and 10 mg kg^−1^ AS1842856 every 2 days on day 17; the corresponding statistical analysis is shown. Representative results from 3 independent experiments are shown (mean ± SD) (n = 5). ∗*P* < .05; ∗∗*P* < .01; ∗∗∗*P* < .001 (unpaired Student *t* test).

We first assessed the combination of Sora with an anti-CD25 antibody in HCC and found that the anti-CD25 antibody notably enhanced the ability of Sora to inhibit the growth of Hepa1-6 xenografts (Figure 7*A*–*C*). Analysis of spleen cells revealed that combination treatment notably increased the number of IFN-γ^+^CD8^+^ T cells, granzyme B^+^CD8^+^ T cells, and Ki67^+^CD8^+^ T cells (Figure 7*D*), indicating that Treg depletion by an anti-CD25 antibody alleviated Sora-induced immunosuppression. Moreover, we performed combination therapy with Sora and the Foxo1 inhibitor AS1842856 (Figure 7*E*). Like the anti-CD25 antibody, AS1842856 also greatly enhanced the therapeutic effects of Sora against Hepa1-6 tumors (Figure 7*F* and *G*). Similarly, combination therapy with Sora and AS1842856 also increased the numbers of IFN-γ^+^CD8^+^ T cells, granzyme B^+^CD8^+^ T cells, and Ki67^+^CD8^+^ T cells among spleen cells (Figure 7*H*). These data collectively highlight the therapeutic potential of combining agents that deplete Treg cells with Sora in HCC.

## Discussion

Epigenetic processes, transport processes, regulated cell death, and TME have been shown to be related to Sora resistance and can be classified as direct or indirect. The lncRNA SNHG1 contributed directly to Sora resistance by activating AKT signaling and its nuclear expression in HCC.^25^ Moreover, miR-494 overexpression directly increased Sora resistance via mTOR pathway activation in HCC, and high miR-494 levels were associated with a lower Sora response in HCC animal models.^26^ Thus, we wanted to explore how Sora influences its own resistance in HCC indirectly. The TME plays a pivotal role in tumor progression because of its large reservoir of immunocompetent cells such as CD8^+^ T cells, NK cells, B cells, and DCs, and immunosuppressive cells such as MDSCs and Treg cells. Recently, a growing number of studies have shown that the infiltration of tumor-associated immunosuppressive cells is important for conferring Sora resistance.^11^^,^^16^^,^^27^ Here we found that Sora significantly increased the proportion of immunosuppressive Treg cells and largely decreased the numbers of antitumor DCs and B cells, thus facilitating the formation of an immunosuppressive TME. It would be interesting to determine whether Sora directly inhibits the infiltration of DCs and B cells. Activated Tregs can secrete cytokines to inhibit the infiltration of DCs and B cells.^28^^,^^29^ Therefore, the decrease in DC and B-cell infiltration might be a result of Sora-induced Treg cell differentiation. Sora can also induce the apoptosis of B cells via reactive oxygen species (ROS) generation, JNK/p38-MAPK activation, and Bax translocation.^30^ In addition, it can inhibit the proliferation of B lymphoblastic cells through the inhibition of PI3K/AKT signaling.^31^ Similarly, Sora inhibits PI3K/AKT and nuclear factor kappa B signaling to reduce DC function by down-regulating DC responsiveness to inflammatory signals.^32^ Therefore, Sora may also directly inhibit the infiltration of B cells and DCs to reprogram the TME.

Interestingly, Treg cells might be recruited to the TME by cytokines such as CCL2 and CCL17 produced by tumor-associated neutrophils, thus promoting the progression of HCC and resistance to Sora.^16^ Here, we are the first to discover that Sora can directly promote Treg cell differentiation via the inhibition of VEGFR/AKT signaling and activation of Foxo1. The effects of an VEGFR inhibitor were similar to those of Sora on Treg cell differentiation. Currently, Sora plus anti-VEGF/VEGFR antibodies are recommended for the clinical management of HCC. Interestingly, administering agents that inhibit Treg cell differentiation could further increase the clinical efficacy of Sora. For example, Foxo1 inhibition abrogated Sora-induced Treg cell differentiation in vitro and increased the therapeutic efficacy of Sora in HCC. Because various targeted therapies such as EGFR-TKIs can inhibit MAPK signaling, it is important to determine whether Treg cell differentiation is promoted and whether inhibiting Treg cell differentiation could increase the clinical efficacy of these targeted therapies.

Collectively, Sora promotes the differentiation but not the proliferation of Treg cells, leading to the expansion of tumor-infiltrating immunosuppressive Treg cells by modulating the VEGFR/ Akt/Foxo1 pathway in CD4^+^ T cells, thus conferring Sora resistance. Inhibiting Treg cell differentiation with an anti-CD25 antibody or the Foxo1 inhibitor AS1842856 increased the therapeutic efficacy of Sora in HCC in vivo, representing a new strategy to increase the clinical efficacy of Sora and potentially other targeted therapies.

## Materials and Methods

### RNA-seq Analysis

Total RNA was isolated and reverse transcribed into cDNA to generate an indexed Illumina library, which was subsequently sequenced at Oebiotech (Shanghai, China) using the BGISEQ-500 platform. High-quality reads were aligned to a mouse reference genome (GRCm38) via Bowtie2. The expression levels of individual genes that were measured via RNA-seq were normalized using the fragments per kilobase million reads using an expectation maximization algorithm. Significant differential expression of a gene was defined as >2-fold expression difference vs the control with an adjusted *P* value <.05. A heatmap was generated via Gene Ontology using Cluster software and visualized with Java Treeview. DEGs were analyzed by Gene Ontology using AMIGO (Barkingside, UK) and DAVID software. The enrichment degrees of the DEGs were analyzed using the KEGG database. The raw sequence data have been submitted to the NCBI Gene Expression Omnibus datasets with accession numbers GSE283371 and GSE280126.

### Mice and Cell Lines

Female C57BL/6J mice (6–8 weeks old) were purchased from Joint Ventures Sipper BK Experimental Animal Co (Shanghai, China). OT-II mice on a C57BL/6J background were purchased from Jackson Laboratory (Farmington, CT). *Foxp3*^*GFP*^ knock-in mice on a C57BL/6J background were provided by Prof Zhijian Cai (Zhejiang University School of Medicine, Hangzhou, Zhejiang, China). The mice were housed in a specific pathogen-free facility, and the experimental protocols were approved by the Animal Care and Use Committee of Zhejiang University School of Medicine (ZJU20240526). Hepa1-6 cells were purchased from the American Type Culture Collection (ATCC, Manassas, VA). The production of Hepa1-6-OVA cells with lentivirus-OVA-GFP (Shiyu Biotechnology Co, Ltd, Hangzhou, Zhejiang, China) was carried out as follows. Hepa1-6 cells were infected with lentivirus in 6-well plates in the presence of 10 μg mL^-1^ polybrene and centrifuged at 1500*g* for 2 hours at 32°C. After 24 hours, the GFP^+^ cells were sorted with a Beckman Coulter DxFLEX flow cytometer (Beckman Coulter Inc, Brea, CA). The OVA overexpression efficiency was confirmed by Western blot analysis. Hepa1-6 and Hepa1-6-OVA cells were cultured in Dulbecco modified Eagle medium supplemented with 10% fetal bovine serum. The cells were incubated at 37°C in a humidified atmosphere composed of 5% CO_2_ and 95% air.

### Human Samples

Human liver tissue samples from HCC patients were obtained from the Affiliated Hangzhou First People’s Hospital, School of Medicine, Westlake University. The Affiliated Hangzhou First People’s Hospital Ethics Committee approved the collection of human samples and patient information. The clinical information of the HCC patients is listed in Table 1.

**Table 1:** Clinical Characteristics of Patients With HCC (n = 30)

Characteristics	No. of patients
Gender	
Male	27
Female	3
Age (y)	
≤50	6
50–75	23
>75	1
Histologic subtype	
Poorly differentiated	10
Moderately differentiated	10
Well-differentiated	10
Pathological subtype	
Tuberous	25
Massive	5

### Tumor Growth Experiments

Individual C57BL/6J mice were injected subcutaneously with 5 × 10^6^ Hepa1-6 cells on day 0. Tumor-bearing mice received 5, 10, or 30 mg kg^−1^ Sora (HY-10201; MedChemExpress, Monmouth Junction, NJ) via intraperitoneal (ip) injection on days 9, 11, 13, and 15. Tumor size was monitored every day by measurement with Vernier calipers.

In vivo depletion of Treg cells was achieved via ip injection of 50 μg anti-CD25 antibody (BE0012; Bio X Cell, West Lebanon, NH) on days 9, 11, 13, and 15. In some experiments, 10 mg kg^−1^ AS1842856 (HY-100596; MedChemExpress) was injected intraperitoneally on days 9, 11, 13, and 15. According to the criteria of the Animal Care and Use Committee of Zhejiang University School of Medicine, the maximal tumor size permitted was 2000 mm^3^, and the tumor sizes in this study did not exceed the maximum size.

TILs were prepared via enzymatic digestion with 1 mg mL^−1^ collagenase IV (CLS-4; Worthington Biochemical Corp, Freehold, NJ) and 0.5 mg mL^−1^ DNase I (DN25; Sigma‒Aldrich) at 37°C for 90 minutes, followed by Percoll (GE Healthcare, Uppsala, Sweden) gradient purification. The isolated TILs were subjected to FC analysis.

### FC Analysis

For surface staining, the cells were stained with fluorescence-conjugated antibodies against surface antigens at room temperature for 20 minutes. To analyze cytokines, the cells were stimulated with Cell Stimulation Cocktail (plus protein transport inhibitors) (00-4975-03; eBioscience, San Diego, CA) for 6 hours, fixed with IC fixation buffer (00-8222-49; Invitrogen, Carlsbad, CA) at room temperature for 20 minutes, permeabilized (00–8333–56l; Invitrogen), and stained with fluorescence-conjugated antibodies against cytokines at room temperature for 30 minutes. Intranuclear staining was performed with fixation/permeabilization buffer (00-5521-00; Thermo Fisher Scientific, Waltham, MA). The stained cells were analyzed using a Beckman Coulter DxFLEX flow cytometer equipped with CytExpert experiment-based software (Beckman Coulter Inc), and the data were analyzed via FlowJo software (TreeStar, Ashland, OR).

To examine cell proliferation, the cells were stained with CFSE (C34554; Thermo Fisher Scientific). The cells were subsequently harvested and washed twice with phosphate-buffered saline. The apoptosis assay was conducted using an Annexin V/PI apoptosis kit (Multi Sciences Biotech, Hangzhou, Zhejiang, China) according to the manufacturer’s instructions.

### In Vitro Treg Cell Differentiation

Naive CD4^+^ T cells were obtained from the spleen and lymph nodes of C57BL/6J mice, *Foxp3*^*GFP*^ mice, or OT-II mice using a mouse naive CD4^+^ T-cell isolation kit (#19765; StemCell, Vancouver, BC, Canada). The sorted naive CD4^+^ T cells were stimulated with plate-bound anti-CD3 (145–2C11, 2 μg/mL; Bio X Cell) and anti-CD28 (PV-1, 2 μg/mL; Bio X Cell) antibodies and polarized into Treg cells with TGF-β1 (10 ng/mL), anti-IFN-γ (10 μg/mL), and anti-IL4 (10 μg/mL) antibodies. In some experiments, the indicated concentrations of Sora (HY-10201; MedChemExpress), Tivozanib (HY-10977; MedChemExpress), VEGF (HY-P7312; MedChemExpress), SC79 (HY-18749; MedChemExpress), AS1842856 (HY-100596; MedChemExpress), U0126 (HY-12031A; MedChemExpress), CP-673451 (HY-12050; MedChemExpress), FLT3-IN-3 (HY-112145; MedChemExpress), and c-Kit-IN-1 (HY-15240; MedChemExpress) were added at the beginning of cell culture. The cells were harvested for real-time PCR analysis or FC analysis on day 3 or for immunoblotting on day 1.

In addition, mouse CD4^+^ or CD8^+^ T cells were isolated with a mouse CD4^+^ T-cell isolation kit (19852) or CD8^+^ T-cell isolation kit (19853) (StemCell Technologies).

### RNA Extraction and Real-time PCR

Total RNA was extracted using TRIzol reagent (9109; TaKaRa, Kusatsu, Shiga, Japan) and reverse transcribed into cDNA using a cDNA synthesis kit (RR047A; TaKaRa) according to the manufacturer’s instructions. Real-time PCR was conducted using SYBR Green Master Mix (RR420; TaKaRa). The following thermal cycling conditions were used for PCR: 1 cycle at 95°C for 30 seconds, followed by 40 cycles at 95°C for 5 seconds and 60°C for 34 seconds. Real-time PCR was performed with an Applied Biosystems 7500 Real-Time PCR System (Waltham, MA). The following primers were used: *mFoxp3*: 5′-*CCTGGTTGTGAGAAGGTCTTCG*-3′ and 5′-*TGCTCCAGAGACTGCACCACTT*-3′; *mIl10*: 5′-*CGGGAAGACAATAACTGCACCC*-3′ and 5′-*CGGTTAGCAGTATGTTGTCCAGC*-3′; *mTgf-beta1*: 5′- *TGATACGCCTGAGTGGCTGTCT*-3′ and 5′- *CACAAGAGCAGTGAGCGCTGAA*-3′.

### Measurement of Cytokine Levels

The levels of TGF-β1 (88-8350-22; Invitrogen) and IL10 (BMS614; Invitrogen) in the cell culture supernatants were measured using ELISA kits according to the manufacturer’s instructions.

### Treg Suppression Assay

Suppression of CD4^+^ or CD8^+^ T-cell proliferation by Treg cells was evaluated by CFSE labeling. Briefly, CFSE (C34570; Invitrogen)-labeled CD4^+^ or CD8^+^ T cells were seeded into a 96-well plate precoated with 2 μg mL^−1^ anti-CD3 antibody plus 2 μg mL^−1^ anti-CD28 antibody with Treg cells at a T cell: Treg cell ratio of 4:1. Five days later, the cells were harvested, and the proliferation of CD4^+^ or CD8^+^ T cells was analyzed using a Beckman Coulter DxFLEX flow cytometer (Beckman Coulter Inc).

### Immunoblotting

Whole cells were washed twice with ice-cold phosphate-buffered saline and lysed with cell lysis buffer (9803; Cell Signaling, Danvers, MA). Proteins in the cell lysates (5–30 μg) were separated via sodium dodecyl sulfate-polyacrylamide gel electrophoresis, transferred onto PVDF membranes (Millipore, Billerica, MA), and probed with primary antibodies against the target proteins.

### Antibodies

Fixable viability dye eFluor 450 (65-0863-14, 1:1000), fixable viability dye eFluor 520 (65-0867-14, 1:1000), APC anti-Foxp3 (17-5773-82, 1:1000), PE anti-CD25 (12-0251-83, 1:1000), PE anti-MHC II (12-5321-82, 1:1000), PE anti-CD11b (12-0112-83, 1:1000), PE anti-CD8 (12-0081-85, 1:1000), APC anti-IFN-γ (17-7311-82, 1:1000), and PE-anti granzyme B (12-8898-80, 1:1000) were purchased from eBioscience (San Diego, CA). PB anti-CD45 (30-F11, 1:1000), APC-cy7 anti-CD4 (100526, 1:1000), APC anti-CD11c (117310, 1:1000), PE-cy5 anti-CD19 (115510, 1:1000), APC anti-F4/80 (123115, 1:1000), APC anti-CD3 (100235, 1:1000), PE anti-NK1.1 (108707, 1:1000), APC anti-Ly6G (164505, 1:1000), APC anti-CD8 (100712, 1:1000), PE anti-Ki-67 (151209, 1:1000), and PE anti-AKT Phospho(Ser473) (606553, 1:1000) were from BioLegend (San Diego, CA). Antibodies against phospho-AKT (Ser473) (4060, 1:2000), AKT (9272, 1:2000), β-actin (4970, 1:2000), p-Smad2 (18338, 1:2000), Smad2 (5339, 1:2000), p-Smad3 (9520, 1:2000), Smad3 (9523, 1:2000), p-ERK (4370, 1:2000), ERK (4695, 1:2000), p-MEK1/2 (9154, 1:2000), MEK1/2 (8727, 1:2000), and GAPDH (2118, 1:2000) were purchased from Cell Signaling Technology (Danvers, MA). Antibodies against phospho-Foxo1(Ser256) (AP0172, 1:1000) and Foxo1 (A13862, 1:1000) were obtained from ABclonal (Wuhan, Hubei, China).

### Multiplex Immunofluorescence Staining and Digital Image Analysis

Multiplex immunofluorescence staining of mouse and human liver tissue samples was performed by Alpha X Biotech Co, Ltd (Beijing, China). Axioscan7 (Zeiss, Oberkochen Germany) was used for imaging. Images were analyzed with HALO software (v3.6; Indica Labs, Albuquerque, NM). Cell segmentation within 11–570 μm^2^ was preliminarily performed with DAPI staining. For density of positive cells/mm^2^, p-AKT^+^CD4^+^ cells or Foxp3^+^CD4^+^ cells in the tumor area were quantified with HALO software by fitting counting algorithms. Correlation analysis between p-AKT^+^CD4^+^ cells or Foxp3^+^CD4^+^ cells was performed via GraphPad Prism 8.0 software (San Diego, CA).

### Statistical Analysis

All the statistical analyses were performed via GraphPad Prism 8.0 software. All the data are expressed as the mean ± standard deviation (SD). Unpaired Student *t* test was used to compare differences between 2 groups. One-way analysis of variance followed by the Newman–Keuls test was used to compare differences among multiple groups. The log-rank test was used for survival analysis, and the Spearman rank-order correlation test was used for Pearson correlation analysis. A difference was considered significant if the *P* value was <.05.
